# Supplementary material for: Synthesis of Retinol-Loaded Lipid Nanocarrier via Vacuum Emulsification to Improve Topical Skin Delivery
Source: Polymers (Basel). 2021 Mar 8;13(5):826. doi: 10.3390/polym13050826 (PMC7962639; doi:10.3390/polym13050826)
Supplement: Supplementary file 1 [file polymers-13-00826-s001.pdf]

Supporting Information

Synthesis of Retinol-loaded Lipid Nanocarrier via Vacuum Emulsification for Improved Topical Skin Delivery

Seung-Hyun Jun [1,]*, Hanul Kim [1], HyeJin Lee [1], Ji Eun Song [1], Sun Gyoo Park [1] and Nea-Gyu Kang [1,]*

(a)

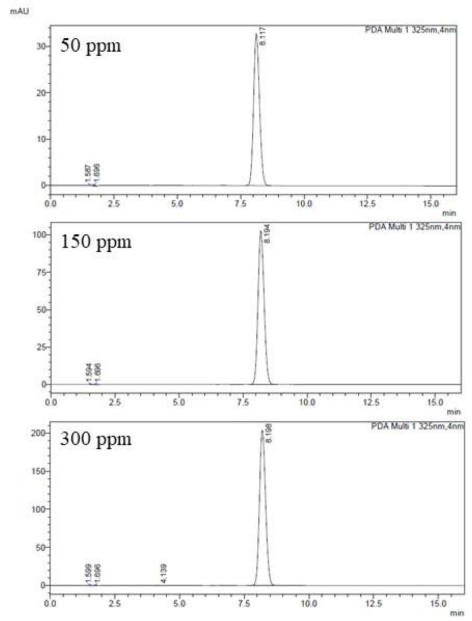

(b)

#	Conc.(Ratio)	MeanArea	Area
1	50	534765	534765
2	150	1682062	1682062
3	300	3333002	3333002

(c)

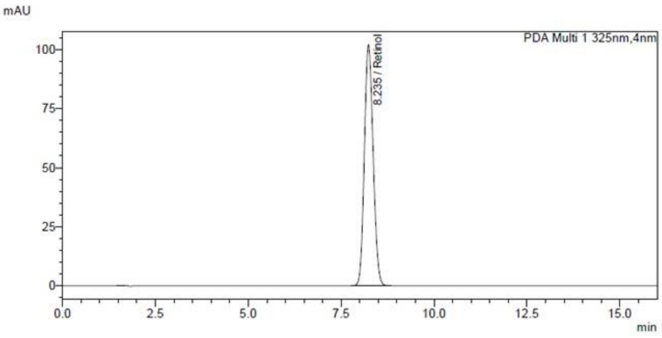

<Quantitative Results>

PDA

ID#	Name	Ret. Time	Area	Height	Conc.	Unit
1	Retinol	8.235	1671730	101993	150.724	mg/L
Total			1671730	101993		

Figure S1. The reference standard solutions of retinol were diluted in three concentrations, 50, 150 and 300 mg/L. The chromatograms are shown in (a). The integrated peak area values are represented in table (b). The chromatogram and peak area of VLN-ROL diluted 200 times are shown in (c). Following the calibration curve of reference standard solution, the concentration was analyzed to over 150 mg/L.
